# Impact of Low‐Dose Cranberry Polyphenols on Gut Microbiota and Circulating Polyphenol Metabolites in Overweight and Obese Individuals (A Randomized Double‐Blind Placebo‐Controlled Clinical Pilot Study)

**DOI:** 10.1002/fsn3.71930

**Published:** 2026-05-29

**Authors:** Maria Jocelyn Chicas Castellon, Min Ji Jang, Maritza Sirven Diaz, Md Ariful Haque, Stephen T. Talcott, Seockmo Ku, Susanne U. Mertens‐Talcott

**Affiliations:** ^1^ Department of Food Science and Technology Texas A&M University College Station Texas USA

**Keywords:** clinical intervention, cranberry, *Eggerthella*, gut microbiota, metabolomics, microbe–metabolite interaction, polyphenols

## Abstract

Cranberry polyphenols reach the colon largely unmetabolized, where they interact with the gut microbiota to generate bioactive metabolites. However, few human studies have examined both the microbial and systemic metabolic responses to cranberry polyphenol intake, particularly in populations with metabolic risk. In this randomized, double‐blind, placebo‐controlled pilot study, 45 overweight or obese adults received either cranberry juice providing 54.5 mg/day of total polyphenols or a placebo for 6 weeks. Serum and urinary polyphenol metabolites were quantified via UPLC–MS/MS, and gut microbiota composition was assessed by 16S rRNA gene sequencing. Cranberry consumption significantly increased serum and urinary concentrations of catechol‐O‐sulfate and 4‐hydroxyhippuric acid, indicating enhanced polyphenol absorption and metabolism. While no overall shift was observed in gut microbial alpha diversity, subgroup analyses revealed increased richness in obese participants and females. Cranberry consumption was associated with taxonomic shifts, including increased abundance of *Anaerostipes*, *Eubacterium hallii* group, and *Eggerthella*. Notably, *Eggerthella* was not detected at baseline but was detected after 6 weeks of cranberry consumption in 8 of 25 participants. Sparse partial least squares analysis showed a positive association between *Eggerthella* and serum 3‐(3‐hydroxyphenyl)propionic acid. These findings suggest a possible microbe–metabolite relationship associated with cranberry intake, although the observed associations are correlative and require further functional validation.

## Graphical abstract

The health‐promoting effects of cranberry are largely attributed to its polyphenol content, which is known to reach the colon in largely unmetabolized form. The cranberry group significantly elevated catechol‐O‐sulfate levels compared to the placebo group. MaAsLin2 analysis revealed a higher relative abundance of *Eggerthella* in the cranberry group at week 6, although this association did not reach statistical significance after multiple testing correction (*p* = 0.00052, *q* = 0.0976). Associations between microbial taxonomic abundance and polyphenol derivatives were further examined, which revealed a notable correlation between *Eggerthella* and specific polyphenol metabolites. These findings suggest a possible microbiome–metabolite succession driven by cranberry polyphenol intake.

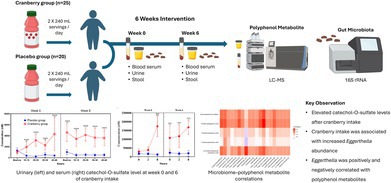

## 1 Introduction

Cranberries ( *Vaccinium macrocarpon* Ait.) are widely recognized for their health‐promoting properties, largely attributed to their high content of polyphenolic compounds, including anthocyanins, flavan‐3‐ols, proanthocyanidins, and flavonols. However, certain cranberry polyphenols—particularly proanthocyanidins—are poorly absorbed in the human small intestine, with more than 90% of the ingested compounds reaching the colon (Lessard‐Lord et al. 2024; Prasain and Barnes 2020).

Consequently, most cranberry polyphenols interact directly with the gut microbiota. Numerous studies have shown that cranberry consumption can alter the composition of the gut microbiota (Blumberg et al. 2016; Cai et al. 2019; Prasain and Barnes 2020).

Although most cranberry polyphenols exhibit low bioavailability in their native form, they can be metabolized by the gut microbiota into smaller compounds that are absorbed into the systemic circulation and subjected to further metabolism (van Duynhoven et al. 2011). These microbial transformations are believed to play a key role in mediating the physiological effects of polyphenols. Therefore, elucidating the interaction between polyphenols and gut microbiota is essential for understanding how these compounds exert their health benefits in the human body.

To date, most studies on this topic have been conducted using animal models or in vitro systems. A recent human clinical study investigated the effects of cranberry extract on gut microbiota and focused on a short 4‐day intervention in healthy adults, examining gut microbial composition and the levels of short‐chain fatty acids in plasma and feces (Lessard‐Lord et al. 2024). Comprehensive human studies are lacking that address the longer‐period effects of cranberry intake, especially in relation to polyphenol metabolism and microbial dynamics within human clinical approaches.

By assessing these outcomes in humans rather than in vitro or in animal models, this study aims to provide valuable phenotypic evidence of how cranberry polyphenols interact with the gut microbiome in vivo. Furthermore, this study focuses on an overweight and obese population, which is of particular interest given the distinctive gut microbiota environment in this population compared to a normal‐weight cohort (Castaner et al. 2018) and potential differences in polyphenol metabolism, also enabling the investigation of individual variability and host‐specific responses to polyphenol intake.

Ultimately, the aim of this study is to (1) characterize the shifts in gut microbiota induced by 6 weeks of cranberry consumption in overweight/obese individuals, (2) identify associations between polyphenol metabolism and specific microbial taxa (e.g., *Eggerthella*). These findings may provide preliminary insights into the potential role of polyphenol‐related microbial responses in the development of functional foods and personalized nutrition strategies.

## 2 Materials and Methods

### 2.1 Study Design and Participants

This study was conducted as a randomized, double‐blind, placebo‐controlled clinical pilot study at Texas A&M University, following receipt of Institutional Review Board (IRB) approval. Forty‐five overweight adults (BMI 28–35 kg/m^2^), aged 18–65 years, with body fat percentages > 18% for males and > 25% for females, were enrolled.

Eligibility criteria were assessed during the screening visit. Exclusion criteria included a recent (within 6 months) history of acute cardiovascular events, stroke, seizures, cancer, hospitalization, or substance abuse. Individuals who smoked (> 1 pack/week) or had liver or renal dysfunction, infectious diseases (hepatitis B/C, HIV), or gastrointestinal disorders (e.g., celiac disease, lactose intolerance) were also excluded. Additional exclusion criteria included pregnancy or lactation, known allergies to cranberry, artificial colors, or flavors, and the use of polyphenol‐rich supplements, prebiotics, or probiotics within 4 weeks prior to screening.

### 2.2 Intervention and Study Procedures

**FIGURE 1:**
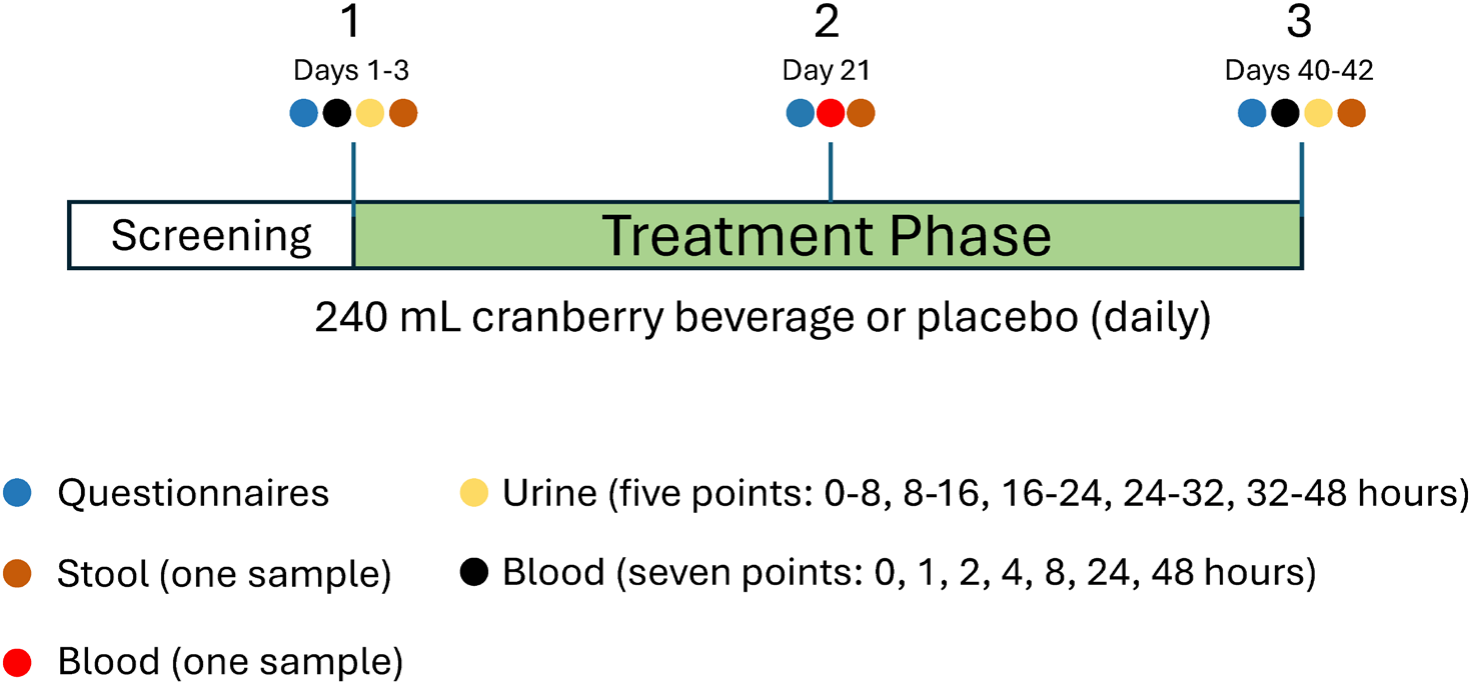
Experimental design used for the human clinical study.

Participants were randomized using a block randomization list (block size = 4) to receive either cranberry juice or a placebo beverage. Both beverages, provided by Ocean Spray Cranberries Inc. (Middleborough, MA, USA), were indistinguishable in color, flavor, viscosity, and overall appearance, and the macronutrient composition was matched between the cranberry juice and the placebo beverage. Each participant consumed two 240 mL servings per day (morning and evening) for 6 weeks (42 ± 2 days) of either cranberry cocktail or placebo beverage (Table 1). Participants attended four in‐person visits, consisting of one screening visit and three study visits at weeks 0 (baseline), 3, and 6 (Figure 1). To minimize variability in polyphenol exposure, participants were instructed to refrain from consuming polyphenol‐rich foods (such as berries, coffee, chocolate, tea, and certain nuts) and dietary supplements for three consecutive days prior to each study visit. A 12 h overnight fast was also required before each visit. On the day of each study visit, participants remained at the clinical site and were provided with standardized low‐polyphenol meals (breakfast, lunch, and dinner) to minimize dietary variability during sample collection. Biological samples were collected and subjected to preparation for downstream analysis.

**TABLE 1:** Concentration of polyphenols, organic acids, and sugars in cranberry and placebo juice (mg/240 mL).

Composition	Cranberry juice	Placebo juice
Total anthocyanins	2.46	N/D
Total phenolic acids	32.46	N/D
Total flavonols	8.82	N/D
Proanthocyanidins	129.92^a^	N/D
	38.44^b^	N/D
Total phenolics^c^	166.34	N/D
Total organic acids	1956	1802
Total sugar	5384	5963

Abbreviation: N/D, non‐detectable.

^a^ Manufacturer‐provided chemical analysis.

^b^ Measured using the 4‐dimethylaminocinnamaldehyde method modified by Brunswick Laboratories.

^c^ Measured using the Folin–Ciocalteu method. Other parameters were determined using HPLC.

### 2.3 Polyphenol Profiling in Cranberry Juice and Biological Samples

#### 2.3.1 Blood and Urine Collection and Preparation for LC–MS/MS Analysis

At weeks 0 and 6, fasting participants provided baseline blood (0 h) and urine samples, followed by consumption of 240 mL of cranberry or placebo juice. Postprandial blood samples were collected at 1, 2, 4, 8, 24, and 48 h, and urine samples were collected at five intervals over 48 h. Blood was allowed to clot, centrifuged (2000*g*, 10 min, 4°C), and serum aliquots (1000 μL) were acidified with formic acid and extracted using 0.1% acidified methanol containing 1 μM ethyl gallate as an internal standard. Samples were vortexed, centrifuged (15,000 rpm, 5 min, 4°C), and filtered using a 3 kDa cutoff membrane before LC–MS/MS analysis. Urine samples were homogenized, acidified with formic acid, and extracted in a 1:1 ratio with acidified methanol containing 1 μM naringenin as an internal standard. After vortexing and centrifugation, the urine samples were filtered through a 0.22 μm filter for LC–MS/MS analysis.

#### 2.3.2 LC–MS/MS Analysis of Cranberry Juice and Biological Samples

The polyphenol compositions of the cranberry juice and biological samples (serum and urine) were analyzed using UPLC–MS with an Ultimate 3000 system coupled to a mass spectrometer (Thermo Fisher Scientific, Waltham, MA). MS analysis was conducted in both positive and negative ion modes with spray voltages of 3500 and 4000 V, respectively. Additional MS settings included sheath gas (20), auxiliary gas (18), and ion transfer tube and vaporizer temperatures (both at 350°C). Selected reaction monitoring (SRM) transitions were determined through direct infusion at 5 μL/min. Samples were maintained at 4°C on an autosampler and injected at volumes of 1, 5, or 10 μL.

Chromatographic separation was carried out on a Synergi Fusion‐RP 80A° column (150 × 2 mm, 4 μm, Phenomenex) at 30°C using a gradient elution with 0.1% formic acid in water (solvent A) and 0.1% formic acid in methanol (solvent B). The gradient profile was 0.5–0 min (5% B), 0–5 min (10%–40% B), 5–7 min (40%–95% B), 7–9.5 min (95% B), and 9.5–10.5 min (95%–10% B), with a flow rate of 0.4 mL/min. Data were acquired using Chromeleon 7.2.10 ES software. As an exploratory step, pharmacokinetic parameters (AUC, *C*_max_, and *T*_max_) were estimated from serum metabolite levels at 0, 2, and 8 h using PKSolver (an Excel add‐in). Due to the limited number of sampling time points (0, 2, and 8 h), pharmacokinetic parameters, including AUC, may not be fully characterized and should therefore be interpreted with caution. Accordingly, these estimates are considered exploratory.

### 2.4 Gut Microbiota Analysis

#### 2.4.1 Stool Sample Collection and DNA Extraction

Participants self‐collected fecal samples on Day 1 and Day 42 of the intervention using stool collection kits (Fisher Scientific, Waltham, MA, USA) and stored samples on ice until delivery. Participants were instructed to avoid laxatives and antacids for 48 h before collection. Samples were delivered within 2 h or stored at freezing temperatures until delivery. Upon receipt, all samples were stored at −80°C until analysis.

#### 2.4.2 16S rRNA Sequencing and Bioinformatics

Gut microbiota composition was assessed using 16S rRNA gene amplicon sequencing targeting the V3–V4 hypervariable regions. PCR amplification was performed using primers 341F and 785R with the HotStarTaq Plus Master Mix Kit (Qiagen, USA). Cycling conditions were as follows: initial denaturation at 95°C for 5 min; 30 cycles of 95°C for 30 s, 53°C for 40 s, and 72°C for 1 min; and a final extension at 72°C for 10 min. Amplicon quality was verified using 2% agarose gel electrophoresis. Equimolar amounts of PCR products were indexed with unique dual barcodes, pooled, and purified using Ampure XP beads. Libraries were prepared according to Illumina protocols and sequenced on a MiSeq platform at MR DNA (Shallowater, TX, USA).

Raw 16S rRNA gene sequences were processed using QIIME 2 (version 2024.10) (Bolyen et al. 2018, 2019). Sequences were demultiplexed using the demux emp‐paired plugin, and primers were removed, chimeras filtered, and reads denoised using the DADA2 denoise‐paired method (Callahan et al. 2016), with trimming parameters set to 0 bp at the start and truncation lengths of 240 bp (forward) and 240 bp (reverse). A feature table and representative sequences were generated and summarized.

Taxonomic classification was performed against the silva database (version 138‐99) at 97% sequence similarity (Quast et al. 2012; Yilmaz et al. 2013). Non‐bacterial ASVs (chloroplasts, mitochondria, and cyanobacteria) and low‐abundance features (< 0.01% of total reads) were excluded. All samples were rarefied to a sequencing depth of 6927 reads based on the minimum depth observed. Alpha diversity was assessed using Chao1, Shannon, and ASV richness indices, while beta diversity was evaluated using Bray‐Curtis and unweighted and weighted UniFrac distances, and visualized via principal coordinates analysis (PCoA).

### 2.5 Statistical Analysis

For LC‐MS data processing, normality was assessed using the Shapiro–Wilk test. If the data were normally distributed, one‐way ANOVA was used to compare concentrations across time points within each treatment group, and two‐way ANOVA was applied to assess treatment effects over time. For non‐normally distributed data, the Mann–Whitney *U* test or the Wilcoxon signed‐rank test was used, as appropriate. Baseline versus week 6 differences between treatments were analyzed using unpaired *t*‐tests or Mann–Whitney *U* tests, depending on normality. Statistical significance was set at *p* < 0.05. All analyses were performed using GraphPad Prism 8.0 (GraphPad Software, San Diego, CA, USA).

For microbiota analysis, alpha diversity was evaluated by treatment groups over time (one‐way ANOVA) or divided into categorical groups based on gender or BMI (overweight or obese) to examine the response to the treatment using a two‐way ANOVA using GraphPad Prism 8.0. Beta diversity was assessed using Bray‐Curtis, weighted UniFrac, and unweighted UniFrac dissimilarity metrics calculated from relative abundance data. Group differences were tested using permutational multivariate analysis of variance (PERMANOVA) and analysis of similarities (ANOSIM), with 999 permutations. All analyses were conducted in QIIME2 (version 2024.10) (Estaki et al. 2020). Differential abundance analysis was conducted using Multivariable Association with Linear Models 2 (MaAsLin2) to identify genus‐level microbial features associated with treatment and time. Relative abundance data were log‐transformed and normalized prior to modeling. Fixed effects included treatment group (cranberry or placebo) and time (week 0 or week 6), with default settings used for other parameters. Statistical significance was evaluated using false discovery rate (FDR)‐adjusted *p*‐values (*q*‐values), with *q* < 0.05 considered statistically significant. Genera with 0.05 ≤ *q* < 0.25 were also reported as exploratory findings to highlight biological trends, consistent with commonly used thresholds in microbiome association studies. Analyses were performed in R (version 4.5.0.) using the MaAsLin2 package (Mallick et al. 2021).

Associations between microbial genera and serum phenolic metabolites were assessed using sparse partial least squares (sPLS) regression implemented in the mixOmics R package (Rohart et al. 2017). This multivariate method integrates high‐dimensional datasets and performs variable selection via Lasso penalization. sPLS was used to identify co‐varying features across the microbiome and metabolome data and to generate correlation heatmaps highlighting key microbe–metabolite relationships.

## 3 Results

### 3.1 Polyphenol Profiling in Cranberry Juice and Biological Samples

Participants consumed two servings of cranberry juice. Each 240 mL serving of cranberry juice contained approximately 27.25 mg of total polyphenols. Phenolic acids (15.05 ± 0.91 mg) were the most abundant class, with p‐hydroxybenzoic acid (4.60 ± 0.08 mg) and *p*‐coumaric acid (3.86 ± 0.46 mg) showing the highest concentrations. Proanthocyanidins (5.89 ± 0.89 mg) were the second most prevalent group, particularly procyanidin A2 (4.89 ± 0.79 mg). Lower levels of anthocyanins (4.24 ± 0.19 mg), flavonols (0.58 ± 0.06 mg), and flavan‐3‐ols (1.49 ± 0.13 mg) were also detected (Table S1).

Of the 36 authentic polyphenol standard compounds, seven metabolites were detected in the biological samples collected after cranberry juice consumption.

Among these, five metabolites were quantifiable based on calibration curves, while 5′‐(3′,4′‐dihydroxyphenyl)‐*γ*‐valerolactone sulfate (detected only in the cranberry group) and quercetin‐3‐O‐*β*‐d‐glucuronide (detected in both the placebo and cranberry groups) were detected below the limit of quantification (< 0.003 nM). Five identified metabolites included two benzoic acid derivatives (3‐hydroxybenzoic acid and 4‐hydroxybenzoic acid), one hippuric acid derivative (4‐hydroxyhippuric acid), one catechol derivative (catechol‐O‐sulfate), and one propionic acid derivative (3‐(3‐hydroxyphenyl) propionic acid). The time (*T*_max_) required to reach peak concentration (*C*_max_) varied between individuals within the same group, ranging from low nanomolar to high micromolar levels, depending on the session, although no significant differences were observed among the groups at baseline or at week 6 (Table S3).

**FIGURE 2:**
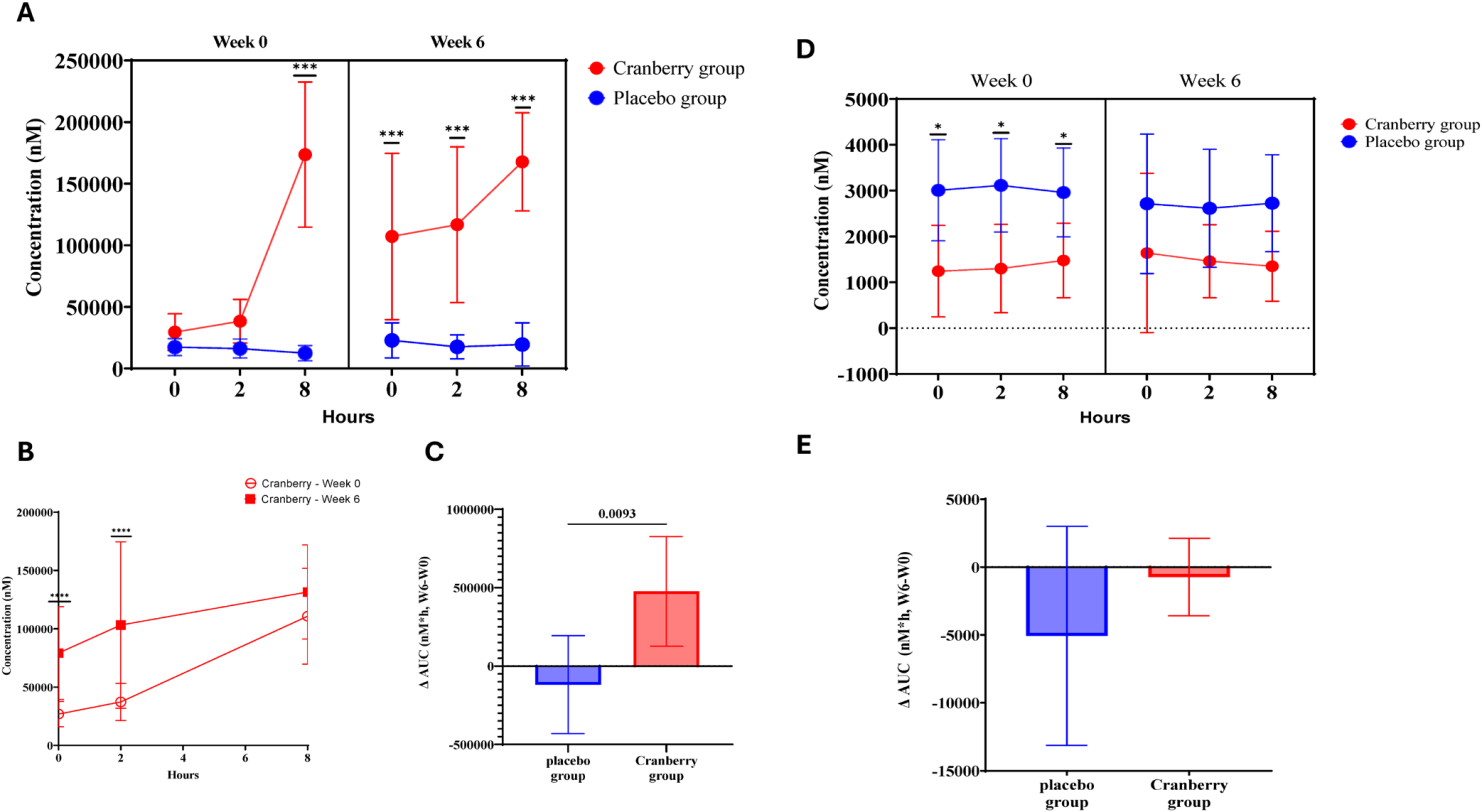
Concentration of (A–C) catechol‐O‐sulfate, (D, E) 3‐(3‐hydroxyphenyl propionic acid) in serum throughout the study period (6 weeks) compared (A, D) among treatment groups (placebo and cranberry), (B) among sessions in the cranberry group, and (C, E) delta (∆) AUC (nM*h) in serum among the treatment groups. Data are presented as mean ± 95% CI. * Significant difference (*p*‐value < 0.05) *** Significant difference (*p*‐value < 0.0001).

Three metabolites (3‐hydroxybenzoic acid, 4‐hydroxybenzoic acid, and 4‐hydroxyhippuric acid) showed no significant differences between treatment groups or between week 0 and week 6. However, significant increases in catechol‐O‐sulfate in the cranberry group during the session (0, 2, and 8 h) indicated greater polyphenol absorption in the cranberry group after 6 weeks of intake of cranberry juice compared to the placebo group (Figure 2A,B). Moreover, when comparing the catechol‐O‐sulfate levels between week 6 and week 0, the cranberry group showed significant increases compared to the placebo group (Figure 2C). For 3‐(3‐hydroxyphenyl)propionic acid, the concentration was significantly higher in the placebo group than in the cranberry group at week 0. After 6 weeks, the concentration decreased in both groups. The decrease was more pronounced in the placebo group, but the difference was not statistically significant (Figure 2D,E).

**FIGURE 3:**
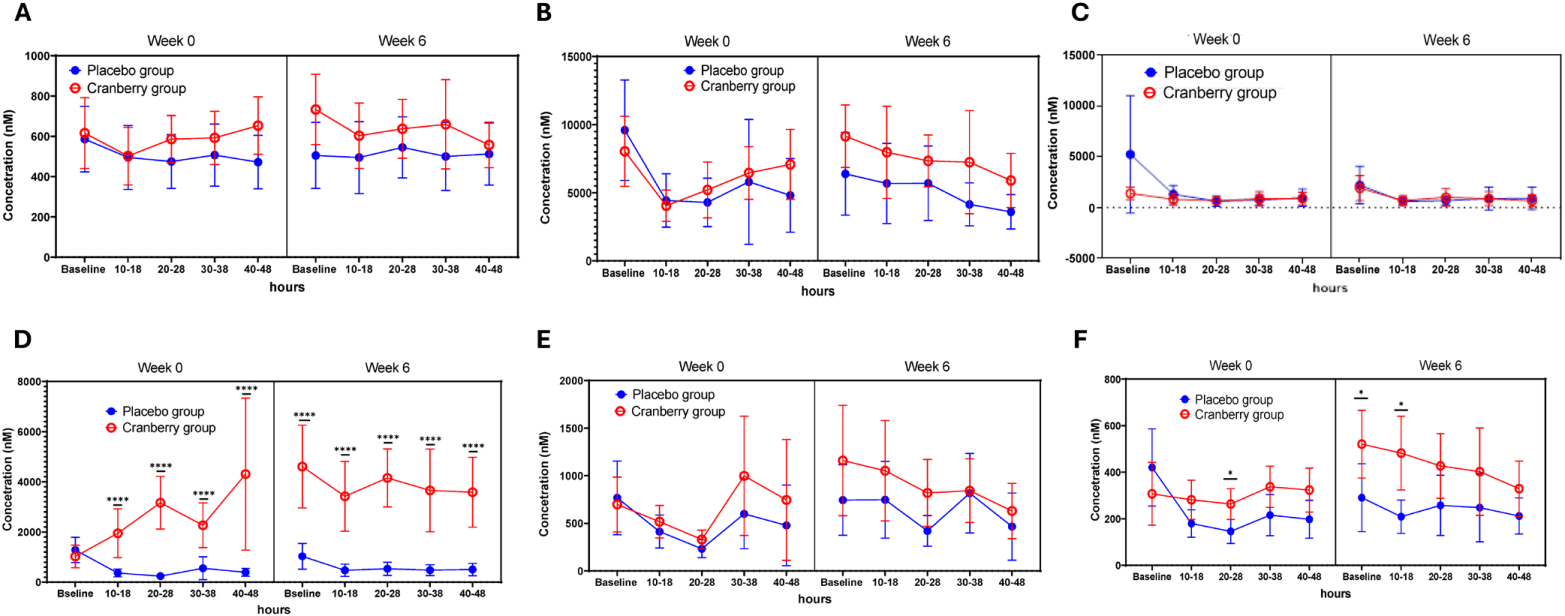
Concentration of polyphenolic metabolites identified in urine over 48 h at the first session (week 0) and final session (week 6) of the treatment intervention. (A) Dihydroferulic acid 4‐*O*‐β‐d‐glucuronide, (B) isoferulic acid 3‐*O*‐*β*‐d‐glucuronide, (C) caffeic acid 4‐*O*‐*β*‐d‐glucuronide, (D) catechol‐*O*‐sulfate, (E) ferulic acid 4‐*O*‐sulfate, and (F) 4‐hydroxyhippuric acid. Data are presented as mean ± 95% CI.

In urine samples, isoferulic acid 3‐*O*‐*β*‐d‐glucuronide was the most abundant metabolite in both groups (Figure 3). At week 6, its concentration was higher in the cranberry group than in the placebo group throughout the 48 h period (Figure 3B). Comparison of its concentration at 20–28 h revealed significant differences in the cranberry group at week 6 (7343.0 nM) and week 0 (5207.7 nM) (*p* < 0.0243) (Table S5). The 4‐hydroxyhippuric acid content at week 0 was higher in the placebo group at the baseline, but the cranberry group showed a significantly higher concentration at 20–28 h (Figure 3F, week 0; Tables S4 and S5). At week 6, the 4‐hydroxyhippuric acid content was higher in the cranberry group throughout the 48 h analysis period after the intervention (Tables S4 and S5). The catechol‐O‐sulfate levels showed the most dramatic difference between the placebo group and the cranberry group (Figure 3D). The levels of catechol‐O‐sulfate increased significantly (*p* < 0.0001) over 48 h after cranberry juice consumption at the first session in the cranberry group compared to the placebo group (Figure 3D). After 6 weeks of cranberry juice treatment, the level of this metabolite was significantly higher in the cranberry group than in the placebo group at all time points from baseline through 48 h (*p* < 0.0001).

### 3.2 Alpha and Beta Diversity

Alpha diversity was represented by three different indices, namely, the amplicon sequence variants (ASVs), Shannon diversity index, and Chao 1. The ASVs indicate species richness, and the Shannon diversity index refers to bacterial community evenness, while Chao 1 represents the abundance of rare bacterial species (Thukral 2017). The mean change (Δ) between day 1 and day 42 was calculated to take into account the initial differences among the groups. None of the alpha diversity indices showed significant differences in mean change between the cranberry and placebo groups. Cranberry intake for 6 weeks did not result in changes in species richness, bacterial community evenness, or the abundance of rare bacterial species compared with baseline (week 0). High values for species richness and bacterial community evenness are associated with a favorable gut microbial profile; therefore, these findings indicate that cranberry intake for 6 weeks may not have produced either beneficial or unfavorable changes in these specific indicators. The alpha diversity analysis results, when stratified by sex and obesity status, showed shifts in only two metrics—ASVs and the Chao1 index—in the obese group, from no significant difference at baseline to a statistically significant difference at week 6. Both metrics significantly increased in the cranberry group compared to the placebo group, suggesting that 6 weeks of cranberry supplementation led to greater species richness and an increased abundance of rare bacterial taxa in obese individuals.

**FIGURE 4:**
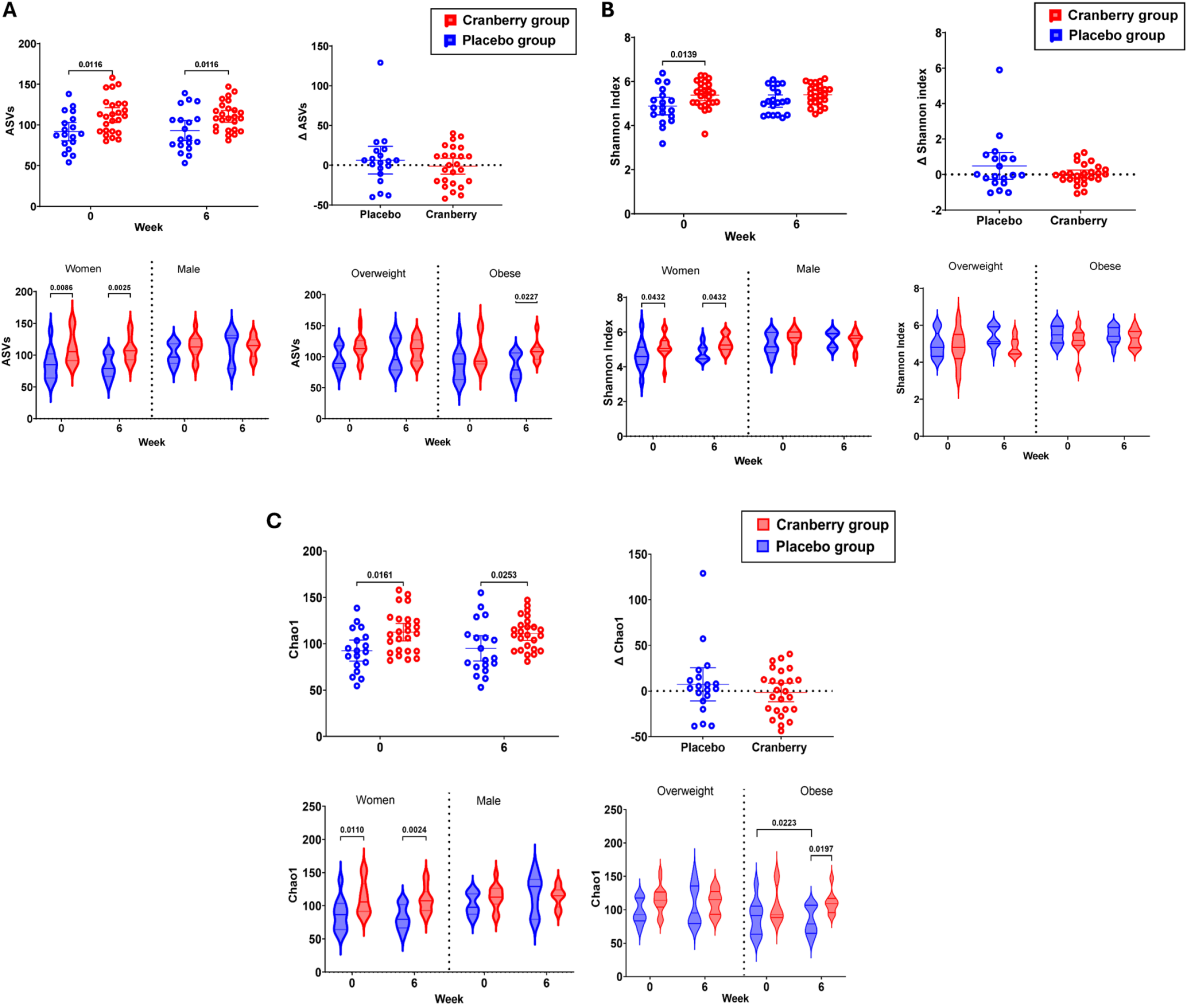
Alpha diversity comparison before and after treatment consumption (placebo or cranberry). (A) Amplicon sequence variants (ASVs) (indicator for species richness), (B) Shannon diversity index (indicator of bacteria evenness), (C) Chao1 (indicator of rare bacteria species abundance) between treatment groups at each session (top left), delta difference between week 0 and week 6 (top right), alpha diversity values stratified by gender (bottom left), and level of obesity (bottom right). The mixed effect model (repeated measures analysis) was used to detect differences among the treatment groups. The delta (week 6—week 0) was calculated to eliminate differences among groups in the initial week (baseline). Results are presented as the mean with 95% CI of the original scores.

Comparison of the male and female participants revealed a more evident difference in the alpha diversity in women between the cranberry and placebo groups. As shown in the violin plots of Figure 4, all three alpha diversity metrics showed statistically significant differences between the cranberry and placebo groups both before and after the 6‐week intervention. However, the differences were more pronounced at week 6 than at baseline. The cranberry group also consistently exhibited higher alpha diversity than the placebo group across all metrics. These findings indicate that the alpha diversity shift induced by cranberry intervention was more prominent in the obese subgroup than in the overweight subgroup and more pronounced in women than in men. The overall direction of the shift suggests an increase in microbial diversity following cranberry supplementation.

Beta diversity, representing the differences between microbial communities, was analyzed using Bray‐Curtis, weighted UniFrac, and unweighted UniFrac dissimilarity metrics. Bray‐Curtis is an abundance‐based dissimilarity index, whereas both UniFrac metrics are phylogenetic beta diversity measures. Bray‐Curtis calculates the distance between microbial communities based on species composition, while UniFrac (unique fraction) metrics consider phylogenetic relationships. Unweighted UniFrac is based on the presence or absence of taxa effectively reflecting rare taxa, whereas weighted UniFrac incorporates their relative abundance, thereby effectively capturing abundant taxa (Xia and Sun 2023).

**FIGURE 5:**
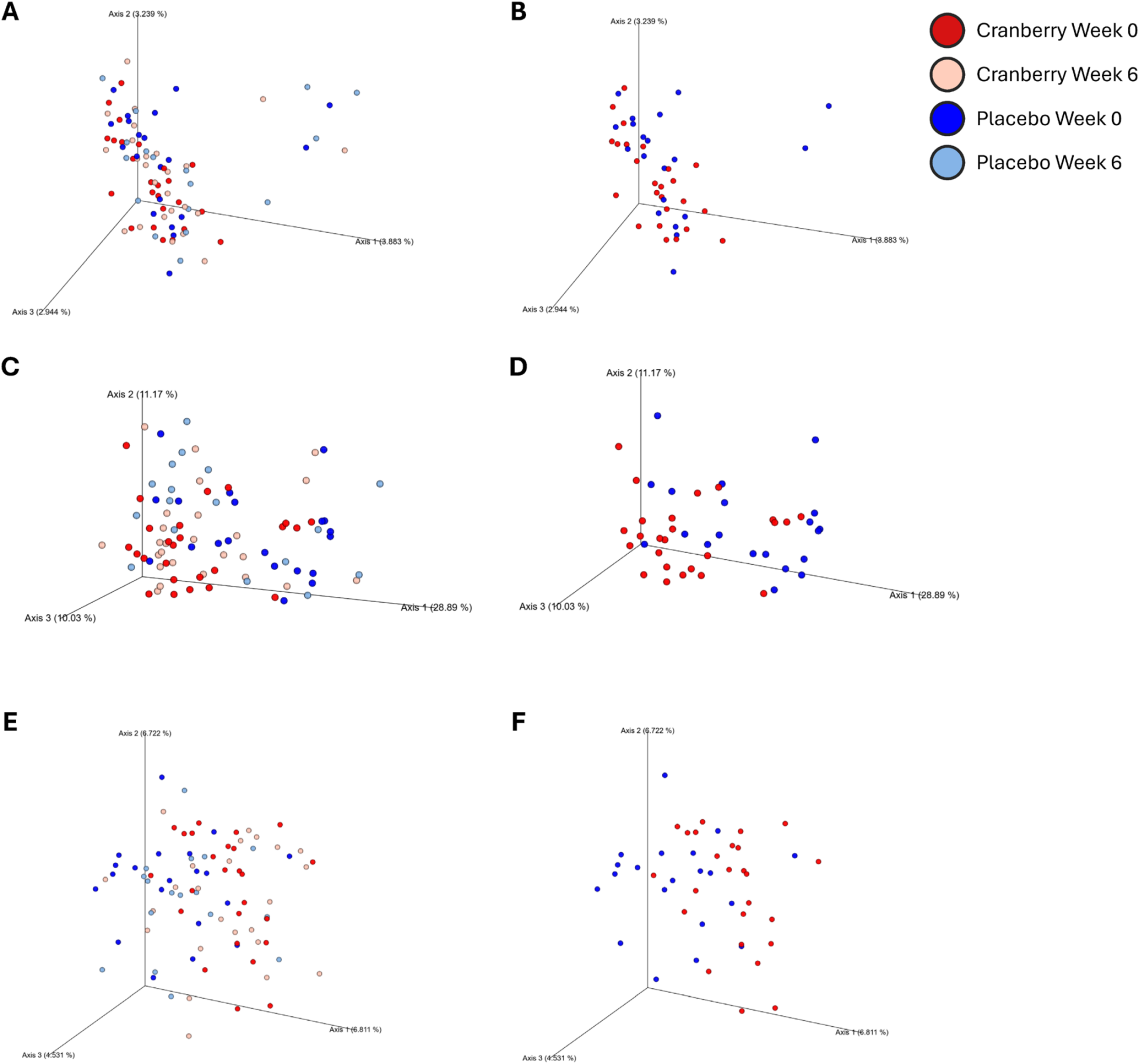
Beta diversity and principal coordinate analysis (PCoA) of (A, B) Bray‐Curtis, (C, D) weighted UniFrac, and (E, F) unweighted UniFrac distances of 16S rRNA genes. Comparison among (A, C, E) all treatment groups and (B, D, F) cranberry and placebo week 6 groups.

ANOSIM showed statistically significant differences across all three beta diversity indices. The low *R* values suggest minimal separation between cranberry week 0, cranberry week 6, placebo week 0, and placebo week 6 (Table S6). In PERMANOVA, Bray‐Curtis was not statistically significant (*p* = 0.296), while the weighted UniFrac (*p* = 0.026) and unweighted UniFrac (*p* = 0.051) showed either significant or borderline results. The lower *p*‐value for the weighted UniFrac indicates that group differences were more detectable when phylogeny and abundance were both considered. The lack of significance in Bray‐Curtis suggests that non‐phylogenetic metrics may be less sensitive to subtle shifts in community structure. The weighted UniFrac consistently showed stronger group separation, which supports the value of phylogenetic metrics in detecting microbiome variation. The beta diversity plots showed no clear clustering across all four groups (Figure 5A,C,E). A separation trend appeared when cranberry week 6 and placebo week 6 were compared (Figure 5B,D,F). The beta diversity results alone do not fully explain the impact of the cranberry intervention. Given the high variability between individuals, the key question that remains is how each host's microbiome responded to the treatment. Although statistical differences were observed, the effect size was small. Further examination is therefore needed to determine how specific taxa change in response to cranberry or placebo treatment.

### 3.3 Taxonomy Analysis

**FIGURE 6:**
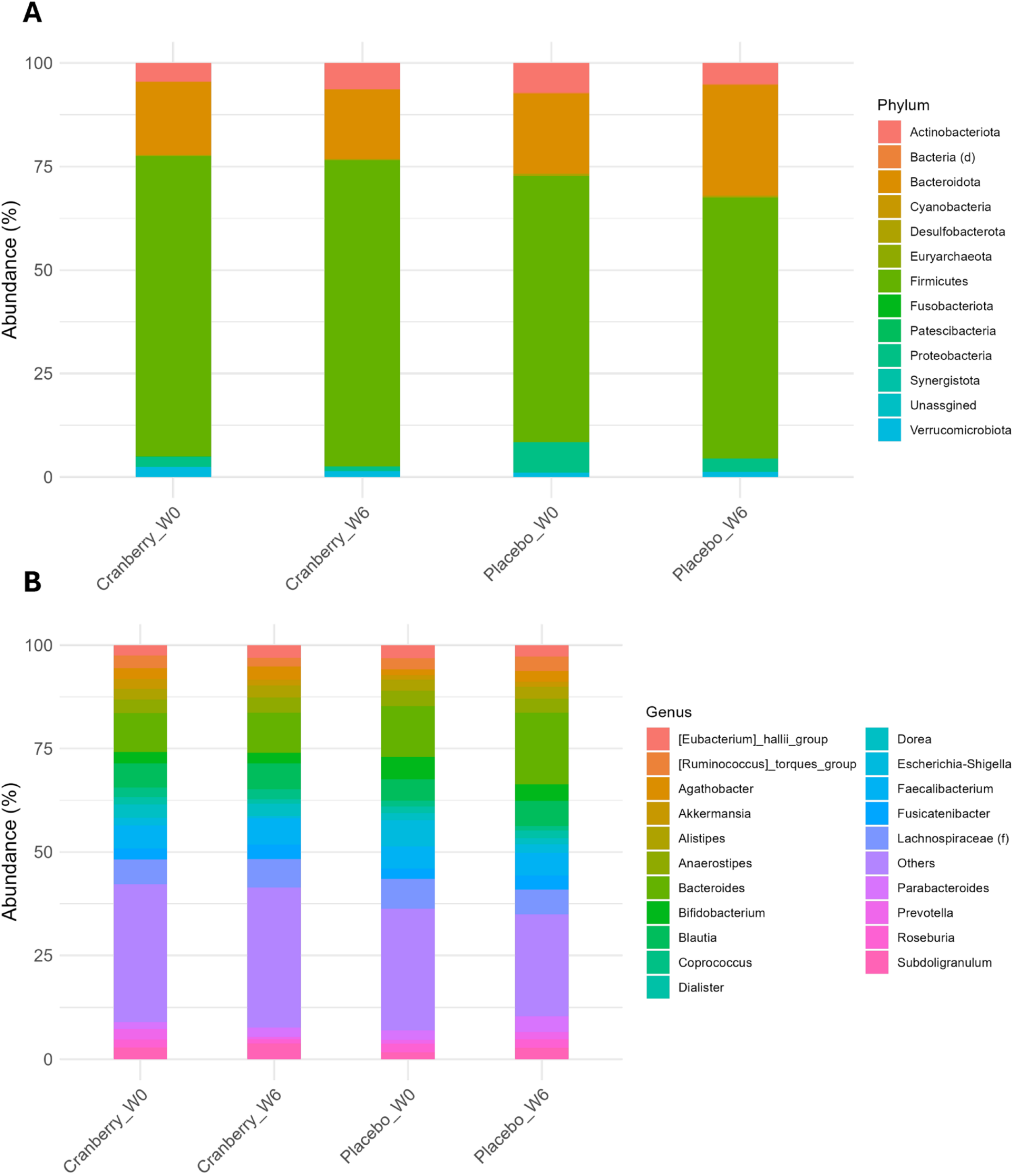
Relative abundance of gut microbiota at the phylum (A) and top 20 genus (B) levels across intervention groups (cranberry, placebo) and time points (week 0, week 6).

At the phylum level, the gut microbiota was dominated by Firmicutes, followed by Bacteroidota, Actinobacteiota, Proteobacteria, and Verrucomicrobiota across all groups. Firmicutes and Actinobacteriota increased in the cranberry‐treated group after 6 weeks but decreased in the placebo group. In contrast, Bacteroidota and Verrucomicrobiota showed the opposite trend, with a relative increase in the placebo group and a decrease in the cranberry group (Figure 6A). These shifts suggest that cranberry supplementation may selectively promote certain beneficial microbial taxa while suppressing others.

At the genus level, *Bacteroides* (9.43%–17.28%), *Blautia* (5.14%–6.26%), *Faecalibacterium* (5.33%–6.39%), *Bifidobacterium* (2.51%–5.42%), and *Anaerostipes* (3.25%–3.71%) were the most abundant across all groups. Six genera among the top 20 showed opposing trends between the cranberry and placebo groups after 6 weeks of treatment: *Anaerostipes*, *Eubacterium hallii* group, and *Coprococcus* increased in the cranberry group but decreased in the placebo group. In contrast, the *Ruminococcus torques* group, *Dialister*, and *Akkermansia* decreased in the cranberry group but increased in the placebo group (Figure 6B).

### 3.4 Comparative Taxonomic Analysis Across Different Groups

MaAsLin2 is a multivariable association framework used to detect differences in the relative abundance of microbial taxa between groups. To control for multiple hypothesis testing and minimize the risk of false positives, MaAsLin2 applies the Benjamini–Hochberg procedure to report adjusted *p*‐values (*q*‐values) (Mallick et al. 2021). MaAsLin2 analysis at the genus level revealed a higher relative abundance of *Eggerthella* (*p* = 0.00052, *q* = 0.0976) and uncultured Coriobacteriales (*p* = 0.00073, *q* = 0.0976) in the Cranberry week 6 group compared with the other groups. However, these associations did not remain statistically significant after false discovery rate (FDR) correction (*q* > 0.05) and should therefore be interpreted as exploratory observations.

A closer examination of *Eggerthella* levels revealed its presence in 8 out of 25 study participants (32%) in the cranberry group. None of those 8 subjects had any detectable *Eggerthella* levels at baseline but exhibited increased relative abundance at week 6 (0.24%–2.96%). In contrast, 4 participants in the placebo group showed detectable levels at baseline (0.23%–0.42%), but 2 had undetectable levels by week 6. Those 2 individuals showed minor increases (from 0% to 0.23% and from 0.18% to 0.46%) (Table 2).

**TABLE 2:** Changes in *Eggerthella* abundance between week 0 and week 6.

Group	Number of subjects with detectable levels of *Eggerthella*	Week 0	Week 6	Trend
Cranberry	8 subjects	All subjects had undetectable *Eggerthella* (0%)	All subjects showed detectable abundance (0.24%–2.96%)	Consistent increase following cranberry intake
Placebo	6 subjects	4 subjects had detectable levels (0.23%–0.42%)	4 subjects decreased to 0%	No consistent trend; partial decrease observed
		2 subjects had 0% and 0.18%	2 subjects showed a slight increase (0% → 0.23%, 0.18% → 0.46%)	

### 3.5 Association Analysis Between Serum Polyphenol Derivatives and Gut Microbial Taxonomy

**FIGURE 7:**
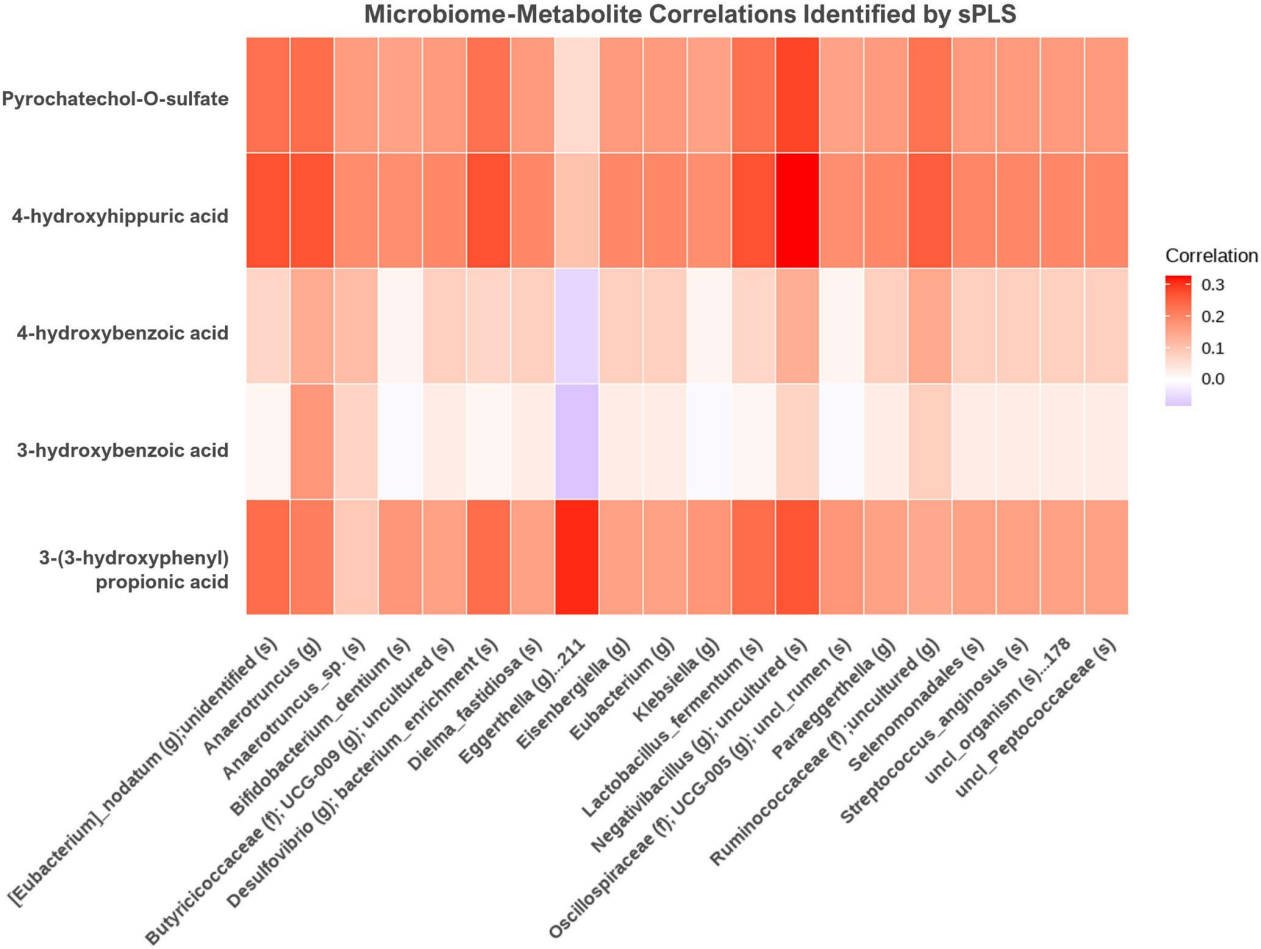
Sparse partial least squares (sPLS) correlations between gut microbial species and serum polyphenol metabolites.

We investigated the associations between microbial taxa and host metabolites using sparse partial least squares (sPLS) regression and the “mixOmics” R package. sPLS is a multivariate statistical method that enables the integration of two high‐dimensional datasets (microbiome and metabolome) while simultaneously performing variable selection. This approach identifies a subset of variables from each dataset that are strongly associated with one another, thereby allowing the detection of biologically interpretable correlation patterns between microbial features and metabolites. In this study, sPLS analysis was used to explore covariance patterns between selected microbial genera and circulating polyphenolic metabolites. This exploratory analysis highlighted several microbe–metabolite associations in the cranberry intervention group. An exploratory correlation pattern was observed for *Eggerthella* in the sPLS analysis. *Eggerthella* was negatively associated with serum 3‐hydroxybenzoic acid and 4‐hydroxybenzoic acid and positively associated with 3‐(3‐hydroxyphenyl)propionic acid (Figure 7). Although these relationships are correlative and should not be interpreted as evidence of direct metabolic activity, they may suggest a potential link between *Eggerthella* and polyphenol metabolism.

## 4 Discussion

### 4.1 Polyphenol Compound Changed Through Cranberry Juice Intervention

This study investigated the effects of cranberry juice consumption over 6 weeks on serum and urine polyphenol metabolites and gut microbiota composition. The daily dose of cranberry polyphenol (54.5 mg/day) administered as juice was lower compared to the dose provided in a previous study using cranberry extract capsules (109.3 mg/day) (Lessard‐Lord et al. 2024). Despite the lower dose used in the present study, the intervention resulted in significant concentration changes for some of the polyphenol derivatives in both serum and urine after the 6 week intervention. Catechol‐O‐sulfate levels increased significantly in both serum and urine samples following cranberry juice intake, indicating a measurable systemic response, even at a modest polyphenol dosage. These changes suggest that cranberry consumption may facilitate the absorption and systemic circulation of certain polyphenol metabolites, particularly catechol‐O‐sulfate. The sustained elevation of catechol‐O‐sulfate for up to 48 h post‐consumption further supports the idea that repeated intake promotes a favorable metabolic profile for polyphenol benefits. Catechol‐O‐sulfate is a low‐molecular‐weight polyphenol metabolite that is found in the plasma following the intake of polyphenol‐rich food and is a well‐known molecule that can penetrate the blood–brain barrier and reach the brain (Carecho et al. 2022). A previous study reported that the intake of polyphenol‐rich mixed berry purée caused an increased catechol‐O‐sulfate level in human plasma, which is consistent with findings in this study (Pimpao et al. 2015). Catechol‐O‐sulfate is a low‐molecular‐weight phenolic metabolite present in human plasma following the consumption of polyphenol‐rich foods, due to absorption in the gastrointestinal tract. In particular, it was reported that catechol‐O‐sulfate appeared 5–9 h after cranberry juice intake, suggesting that its formation is mediated by gut microbiota (Feliciano et al. 2016). This metabolite has been associated with cardiovascular protective effects by mitigating oxidative stress (Dias‐Pedroso et al. 2019; Yoshizumi et al. 1996), and it has also been shown to activate molecular defense mechanisms in dopaminergic neurons under toxic stress conditions (Carecho et al. 2022). In the present study, cranberry consumption was associated with increased circulating catechol‐O‐sulfate levels. Although catechol‐O‐sulfate has been linked to cardiovascular and neuroprotective activities in prior studies, the present was not designed to evaluate these physiological outcomes directly.

An additional limitation of the present analytical approach is that several metabolites were detected at levels below the limit of quantification or could not be fully characterized. The use of high‐resolution mass spectrophotometry in future studies would improve the structural annotation and detection of low‐abundance cranberry‐derived metabolites and enable a more comprehensive assessment of polyphenol bio transformation.

### 4.2 Changes in Gut Microbiota Through Cranberry Juice Intervention

While no statistically significant difference was detected in delta alpha diversity (week 6—week 0) between the cranberry and placebo groups overall, subgroup analyses revealed distinct trends. Notably, alpha diversity significantly increased among obese participants in the cranberry group (compared to overweight participants) and as well in women compared to men. Previous studies have associated increased alpha diversity with a more favorable and resilient gut microbial environment (Kundu et al. 2017; Zhou et al. 2025). These findings suggest that cranberry supplementation even at a low dose may promote beneficial host‐dependent shifts in gut microbial diversity, particularly among women and individuals with obesity. This differential response to cranberry intervention across groups suggests that identical dietary interventions may elicit variable gut microbiome responses depending on individual characteristics as previously observed (Alsuhaibani et al. 2022; Jensen‐Kroll et al. 2025). While alpha diversity did not significantly change between treatment groups overall, there was a significant increase in women and obese individuals, which may imply that benefits of cranberry supplementation may be more pronounced in women and obese individuals. Demographic variables may act as modulators of gut microbiota composition in response to specific dietary interventions. Recent studies have emphasized the importance of personalized nutrition, aiming to tailor dietary recommendations based on phenotypic and health‐related differences among individuals (Hu et al. 2024). Although the present findings alone are not sufficient to establish concrete clinical guidelines, they provide preliminary evidence that supports the rationale for exploring personalized dietary strategies for elevating gut microbial diversity.

The beta‐diversity results revealed statistical differences among the four groups (week 0 placebo, week 6 placebo, week 0 cranberry, and week 6 placebo). However, small *R* values limit the detection of overall difference in microbial composition between groups. A previous study (Lessard‐Lord et al. 2024) reported contrasting results, where the intake of cranberry extract containing twice the amount of polyphenols compared to the treatment used in this study led to significant differences in beta diversity after just 4 days of supplementation. In that study, cranberry intake explained 18.5% of the variance in the microbial community composition, highlighting an inconsistency with the present finding. One clear limitation of the previous study was the absence of a placebo‐controlled design. To establish a more reliable consensus on how cranberry polyphenols influence the human gut microbiome, further human clinical studies with rigorous controls and diverse dosages are needed.

### 4.3 Microbial Composition Changes After Cranberry Juice Intervention

At the genus level, six bacterial genera showed opposing trends between the cranberry and placebo groups following the 6 weeks intervention. Among the taxa that increased in relative abundance after 6 weeks cranberry intervention were *Anaerostipes*, *Eubacterium hallii* group, and *Coprococcus. Anaerostipes* is a genus known to produce butyrate (Fu et al. 2019). Butyrate plays a critical role in maintaining intestinal epithelial cell integrity, partly through the regulation of tight junction proteins, and it has been reported to have cancer‐preventive effects (Liu et al. 2018). A reduction in butyrate‐producing species, including *Anaerostipes*, has also been associated with Crohn's disease, type 2 diabetes, and ulcerative colitis (Liu et al. 2024). A previous study reported a decrease in the relative abundance of *Coprococcus* in the gut microbiota of patients with early hepatocellular carcinoma (Ren et al. 2019), suggesting a potential association between reduced *Coprococcus* abundance and liver carcinogenesis. The *Eubacterium hallii* group, now reclassified as *Anaerobutyricum hallii*, is another butyrate‐producing genus that has a known relationship with improved insulin sensitivity (Shetty et al. 2018; Udayappan et al. 2016).

Following the 6 week placebo intervention in this study, *Ruminococcus_torques_group*, *Dialister*, and *Akkermansia* increased in relative abundance. The *Ruminococcus torques* grouphas been identified as a mucin‐degrading bacterial taxon, and its increased abundance has been associated with inflammatory bowel disease (IBD) through a potential contribution to epithelial barrier dysfunction and mucosal inflammation (Tran et al. 2023). Mucin‐degrading bacteria, such as the *Ruminococcus torques* group, can function as keystone species by liberating carbohydrates that support the growth of other commensals within the gut ecosystem. However, dysregulated or excessive mucin degradation has been associated with impaired intestinal barrier integrity and increased mucosal inflammation, particularly in the context of IBD (Schaus et al. 2024). Previous studies have associated the genus *Dialister*, which increased in the placebo group after 6 weeks, with oral inflammatory conditions such as periodontitis and with spondyloarthritis characterized by subclinical intestinal inflammation (Demirci 2021; Tito et al. 2017).

*Akkermansia* is another well‐known mucin‐degrading genus, with *Akkermansia muciniphila* being the most extensively studied species (González et al. 2023). The presence of *A. muciniphila* has been associated with beneficial effects on host metabolism, mucosal immune regulation, and intestinal barrier integrity, making it a prominent candidate for next‐generation probiotics (González et al. 2023). Although both *Akkermansia* and *Ruminococcus* are capable of degrading mucin, they are associated with distinct impacts on host health. The *Ruminococcus torques* group has been linked to increased intestinal permeability and inflammation in patients with IBD, while *Akkermansia muciniphila* has been shown to strengthen the epithelial barrier function and shows an inverse association with inflammatory bowel disease (IBD) severity (González et al. 2023; Henke et al. 2019). Although the abundance of the beneficial genus *Akkermansia* increased in the placebo group at week 6, genera that increased in the cranberry group were more consistently associated with health‐promoting effects, whereas those that increased in the placebo group tended to be linked with less favorable health profiles.

Importantly, the present findings are based on taxonomic and metabolite‐level associations and do not establish a mechanistic role for *Eggerthella* in cranberry polyphenol metabolism. Future studies incorporating ex vivo fecal fermentation models, targeted gene profiling, shotgun metagenomics, or metatranscriptomic approaches will be necessary to determine whether *Eggerthella* directly participates in the degradation or transformation of cranberry‐derived polyphenols.

### 4.4 Correlation Between Serum Polyphenol Metabolite and Gut Microbial Taxonomy

MaAsLin2 analysis suggested a trend toward higher relative abundance of *Eggerthella* and uncultured *Coriobacteriales* in the cranberry week 6 group. Taxonomic profiling at the individual level further revealed an increasing trend for *Eggerthella* in the cranberry group (8/25 subjects: increase), while the placebo group showed a decreasing trend (4/20 subjects: decrease; 2/20 subjects: increase). These findings suggest that 6 weeks of cranberry consumption may be associated with the emergence or increased detectability of *Eggerthella* in a subset of participants, although considerable inter‐individual variability was observed.

While the trend did not reach statistical significance, the emergence of *Eggerthella* in a subset of subjects exclusively in the cranberry group may reflect an exploratory response to dietary intervention that warrants further study.

Previous clinical studies have demonstrated that consumption of polyphenol‐rich red wine and dealcoholized red wine led to a significant increase in *Eggerthella lenta* and *Bifidobacterium* levels compared to the baseline (Nash et al. 2018). Another study conducted ex vivo fecal fermentation with rutin and genistein, which are polyphenol compounds, and found that the relative abundance of *Eggerthella* was significantly higher in the fermented samples supplemented with polyphenol derivatives indicating the polyphenols can foster *Eggerthella* growth (Jensen‐Kroll et al. 2025). This observation is consistent with the present finding of an increased abundance of *Eggerthella* following the consumption of a polyphenol‐rich product. However, *E. lenta* is also recognized as a pathogenic bacterium known to cause bacteremia (Jiang et al. 2021). In patients with bronchiectasis, an elevated level of *E. lenta* in the gut microbiota has been associated with intensified lung infections. This has been attributed to *E. lenta* ‐derived tauroursodeoxycholic acid (TUDCA), which downregulates AMPK activity (Wang et al. 2025). Although *E. lenta* is primarily reported as a risk factor, some reports suggest a context‐dependent role. For example, a study involving elderly individuals found decreased levels of *Eggerthella* in a liver cancer group compared to healthy controls (Zhang et al. 2023), possibly reflecting the hepatoprotective properties of bile acids, such as TUDCA, that are produced by *Eggerthella*. As members of the *Eggerthella* genus are also part of the normal gut microbiota, it is plausible that *E. lenta*, while potentially pathogenic in some contexts, may also have beneficial or regulatory roles in others. This duality highlights the importance of further investigation aimed at determining whether the *Eggerthella* strains introduced or promoted by cranberry intake have positive or negative physiological effects.

A limitation of this study is that 16S rRNA gene sequencing was used, which restricts taxonomic resolution to the genus level and does not allow strain‐level identification of *Eggerthella*. As a result, strain‐specific functional capacity and potential effects on host health could not be determined. This also limits distinguishing between potentially beneficial and opportunistic strains, including species such as *Eggerthella lenta* , which has been associated with opportunistic infections. Therefore, the biological implications of increased *Eggerthella* abundance should be interpreted with caution. In addition, the present study does not provide direct evidence of polyphenol degradation pathways, and the observed associations between *Eggerthella* and polyphenol metabolites remain correlative. Future studies using shotgun metagenomic sequencing or other advanced approaches will be required to achieve strain‐level resolution and to clarify the functional role and safety implications of *Eggerthella* in polyphenol metabolism. *Coriobacteriales incertae sedis*, an order within *Actinobacteriota*, is currently poorly characterized, but other members within the sample clade, such as *Coriobacteriaceae* and *Eggerthellaceae*, are well known as polyphenol‐degrading bacteria (Rodríguez‐Daza et al. 2020). In support of this, Rodríguez‐Daza et al. (2020) showed that obese mice fed cranberry powder exhibited a five‐fold increase in *Eggerthella*ceae and a 10‐fold increase in Coriobacteriales when compared to obese mice fed a high‐fat, high‐sucrose diet. The increased composition of *Coriobacteriales incertae sedis* and *Eggerthella* in our cranberry week 6 group is consistent with this finding and cranberry‐associated shifts in selected taxa. However, because this study relied on 16S rRNA sequencing and correlative metabolite analyses, functional implications and effects on polyphenol biotransformation cannot be established directly. Future studies incorporating functional approaches, such as ex vivo fermentation models, will be necessary to determine whether taxa such as *Eggerthella* and *Coriobacteriales incertae* sedis are directly involved in polyphenol metabolism and to elucidate their potential mechanistic roles. However, direct experimental evidence linking these taxa to polyphenol metabolism remains limited, highlighting the need for further targeted investigation. In this study, sPLS analysis revealed an association between *Eggerthella* abundance and serum polyphenol metabolites following cranberry intake. The direct conversion of 3‐hydroxybenzoic acid and 4‐hydroxybenzoic acid to 3‐(3‐hydroxyphenyl)propionic acid is unlikely, as no established biochemical pathway supports this transformation. Instead, data suggest that the metabolic flux, which typically proceeds toward 3‐hydroxybenzoic acid and 4‐hydroxybenzoic acid, is redirected predominantly toward the biosynthesis of 3‐(3‐hydroxyphenyl)propionic acid in the presence of *Eggerthella*. The observed patterns suggest that *Eggerthella* abundance may be associated with variation in circulating polyphenol metabolites; however, these associations are correlative and do not establish a causal or functional role in polyphenol metabolism. According to a previous study, the concentration of 3‐(3‐hydroxyphenyl)propionic acid increased markedly from 0.9 μmol/L to 21.56 μmol/L within 5 h during a 24 h fecal slurry fermentation (Bresciani et al. 2021). In contrast, 3‐hydroxybenzoic acid and 4‐hydroxybenzoic acid showed only slight increases during the 24 h fermentation, with concentrations changing from 1.98 to 2.81 μmol/L and from 1.07 to 2.57 μmol/L, respectively (Bresciani et al. 2021).

Taken together with prior reports, these findings are consistent with the interpretation that 3‐(3‐hydroxyphenyl)propionic acid can arise from gut microbial metabolism of polyphenols, whereas the production of 3‐ and 4‐hydroxybenzoic acid appears to be limited. The elevated level of 3‐(3‐hydroxyphenyl)propionic acid could therefore serve as an indicator that the gut microbiota is actively involved in polyphenol metabolism. In the present study, *Eggerthella* emerges as one of the genera associated with 3‐(3‐hydroxyphenyl)propionic acid in the integrative analysis. This observation should be considered hypothesis‐generating, as the study design does not permit mechanistic attribution or confirmation of metabolic function.

Collectively, the findings from this study suggest that 6 weeks of cranberry juice consumption, even at a low‐dose polyphenol level, can lead to measurable increases in systemic polyphenol metabolites, such as catechol‐O‐sulfate, along with alterations in gut microbiota composition. Importantly, the increased abundance of butyrate‐producing genera, such as *Anaerostipes* and *Eubacterium hallii* group, and the emergence of *Eggerthella* and *Coriobacteriales incertae sedis* in the cranberry group indicate a potential microbiotal shift that may favor microbial polyphenol biotransformation. The observed correlation between *Eggerthella* abundance and serum 3‐(3‐hydroxyphenyl)propionic acid levels further supports the involvement of specific taxa in modulating host polyphenol metabolism. Although interindividual variability and the lack of statistical significance in some comparisons limit the strength of interpretation, the consistent directional trends provide mechanistic insight into microbiota‐mediated metabolic responses to polyphenol intake. These observations highlight the potential of using cranberry polyphenols to modulate gut microbiota composition and function in a host‐specific manner and warrant further investigation in larger and more targeted studies.

In addition, microbiota samples were collected only at baseline and week 6, thereby limiting the ability to capture intermediate temporal dynamics of microbial responses. Future studies incorporating additional sampling time points (e.g., mid‐intervention) will be necessary to more fully characterize the temporal trajectory of microbiota adaptation.

## 5 Conclusions

This study provides insight into the interplay between cranberry polyphenols and the gut microbiota by simultaneously profiling serum polyphenol metabolites and microbial composition in human participants following 6 weeks of cranberry juice consumption. This study integrates both polyphenol‐related metabolomic and microbiome data to examine how cranberry‐derived polyphenols influence, and are influenced by, gut microbial communities. The co‐variation of *Eggerthella* and 3‐(3‐hydroxyphenyl)propionic acid suggests a possible microbe–metabolite association during cranberry polyphenol metabolism in the human gut. While these findings contribute to the groundwork for future mechanistic investigations, these do not establish causality or physiological consequences of these microbial metabolic changes. Although members of the *Eggerthella* genus have been implicated in polyphenol metabolism, some species, including *Eggerthella lenta* , have also been associated with opportunistic infections. Therefore, the biological significance of increased *Eggerthella* abundance should be interpreted with caution, and further strain‐ and function‐level investigations will be required to clarify its role, particularly using approaches such as shotgun metagenomics.

Notably, this study demonstrates that consumption of a low dose of cranberry polyphenols over a 6‐week period may modulate gut microbial composition and influence circulating polyphenol metabolite profiles. Furthermore, there appears to be a trend suggesting that these effects were more pronounced among female participants and individuals with obesity.

However, the observed associations, particularly those involving *Eggerthella*, should be interpreted with caution, as they are correlative and do not establish a mechanistic role in polyphenol metabolism. Further research is therefore needed to elucidate the functional role of *Eggerthella* and to evaluate its clinical relevance in the health‐promoting effects of cranberry consumption.

In particular, future studies using high‐resolution mass spectrometry together with functional microbiome approaches, such as ex vivo fermentation and gene‐level analyses, will be important to clarify the pathways and taxa involved in cranberry polyphenol metabolism.

## Author Contributions

**Min Ji Jang:** methodology, formal analysis, writing – original draft, investigation, visualization. **Maria Jocelyn Chicas Castellon:** methodology, formal analysis, investigation, writing – original draft, visualization. **Maritza Sirven Diaz:** formal analysis, investigation. **Md Ariful Haque:** investigation, formal analysis. **Stephen T. Talcott:** investigation, formal analysis, supervision. **Seockmo Ku:** investigation, formal analysis, supervision, writing – review and editing, writing – original draft. **Susanne U. Mertens‐Talcott:** study design and supervision, writing – original draft, writing – review and editing, investigation, formal analysis, resources.

## Funding

This work was supported by Ocean Spray Cranberries Inc.

## Conflicts of Interest

The authors declare no conflicts of interest.

## Supporting information


**Table S1:** MS parameters are used for the identification of metabolites derived from cranberry polyphenols present in cranberry and placebo beverage.
**Table S2:** MS parameters are used for the identification of metabolites derived from cranberry polyphenols present in serum and urine samples.
**Table S3:** Pharmacokinetic parameters of serum polyphenol metabolites following cranberry or placebo juice consumption.
**Table S4:** Urinary metabolite concentrations (nM) across post‐consumption time windows (10–48 h) at week 0 and week 6 of placebo juice intervention.
**Table S5:** Urinary metabolite concentrations (nM) across post‐consumption time windows (10–48 h) at week 0 and week 6 of cranberry juice intervention.
**Table S6:** ANOSIM and PERMANOVA statistics for gut microbiota beta diversity across Bray‐Curtis, Weighted, and Unweighted UniFrac metrics.

## Data Availability

The data that support the findings of this study are available from the corresponding author upon reasonable request.
