# Supplementary material for: Impact of Low‐Dose Cranberry Polyphenols on Gut Microbiota and Circulating Polyphenol Metabolites in Overweight and Obese Individuals (A Randomized Double‐Blind Placebo‐Controlled Clinical Pilot Study)
Source: Food Sci Nutr. 2026 May 29;14(6):e71930. doi: 10.1002/fsn3.71930 (PMC13239935; doi:10.1002/fsn3.71930)
Supplement: Supplementary file 1 — Table S1: MS parameters are used for the identification of metabolites derived from cranberry polyphenols present in cranberry and placebo beverage. Table S2: MS parameters are used for the identification of metabolites derived from cranberry polyphenols present in serum and urine samples. Table S3: Pharmacokinetic parameters of serum polyphenol metabolites following cranberry or placebo juice consumption. Table S4: Urinary metabolite concentrations (nM) across post‐consumption time windows (10–48 h) at week 0 and week 6 of placebo juice intervention. Table S5: Urinary metabolite concentrations (nM) across post‐consumption time windows (10–48 h) at week 0 and week 6 of cranberry juice intervention. Table S6: ANOSIM and PERMANOVA statistics for gut microbiota beta diversity across Bray‐Curtis, Weighted, and Unweighted UniFrac metrics. [file FSN3-14-e71930-s001.docx]

**Supplement**

**Table S1.** MS parameters are used for the identification of metabolites derived from cranberry polyphenols present in cranberry and placebo beverage.

| **Metabolites** | **[M-H]^-^** | **MS/MS fragments** | Concentration  (mg/240 ml) |
| --- | --- | --- | --- |
| **Phenolic acids** |  |  | **15.05** **± 0.91*** |
| Benzoic acid | 65.071 | 65.07,77.05,92.04 | 0.97 ± 0.14 |
| 3,4-Dihydroxybenzoic acid (Protocatechuic acid) | 81.054 | 81.05,91.04,109.04 | 1.47 ± 0.11 |
| Gallic acid | 79.113 | 79.11,97.04,125.11 | 0.35 ± 0.02 |
| Vanillic acid | 107.97 | 107.97,123.04,152.04 | 0.02 ± 0.01 |
| *p-coumaric acid* | 93.071 | 93.07,117.00,119.04 | 3.86 ± 0.46 |
| Caffeic acid | 122.042 | 122.04,135.04,164.07 | 2.05 ± 0.06 |
| Chlorogenic acid | 85.071 | 85.07,161.13,191.02 | 1.48 ± 0.01 |
| *p-hydroxybenzoic acid* | 65.054 | 65.05,90.97,93.04 | 4.60 ± 0.08 |
| Sinapic acid | 149.042 | 149.04,192.97,207.96 | 0.04 ± 0.00 |
| Caffeic acid 4-glucuronide | 135.054 | 135.05,175.04,179.05 | 0.22 ± 0.02 |
| **Flavan-3-ols** |  |  | **1.49 ± 0.13** |
| Catechin | 203.071 | 203.07,204.97,245.04 | 0.39 ± 0.04 |
| (-) epicatechin | 205.071 | 205.07,109.13,124.75 | 1.10 ± 0.09 |
| **Flavonols** |  |  | **0.58 ± 0.06** |
| Quercetin-3-glucoside | 255.125 | 255.13,271.08,299.77 | 0.40 ± 0.04 |
| Quercetin 3-glucuronide | 151.042 | 151.04,179.04,301.05 | 0.01 ± 0.00 |
| Rutin | 254.97 | 254.97,271.04,300.04 | 0.17 ± 0.02 |
| **Anthocyanins** |  |  | **4.24 ± 0.19** |
| Cyanidin-3-glucoside | 255.083 | 255.08,284.13,285.13 | 0.04 ± 0.00 |
| Cyanidin-3-Arabinoside | 255.054 | 255.05,284.11,357.08 | 0.22 ± 0.03 |
| Peonidin-3-Arabinoside | 298.155 | 298.16,299.16,385.14 | 0.40 ± 0.01 |
| Peonidin-3-Galactoside | 191.042 | 191.04,298.13,415.24 | 1.88 ± 0.07 |
| [Malvidin 3-arabinoside](https://pubchem.ncbi.nlm.nih.gov/compound/Malvidin-3-arabinoside-cation) | 131 | 131.00,149.00,330.16 | 0.90 ± 0.05 |
| Delphinidin 3-O-arabinoside | 271.083 | 271.08,300.08,301.13 | 0.80 ± 0.02 |
| **Proanthocyanidins** |  |  | **5.89 ± 0.89** |
| Procyanidin B2 | 289.208 | 289.21,407.14,425.21 | 1.01 ± 0.10 |
| [Procyanidin A2](https://www.google.com/search?rlz=1C1AVFC_enUS922US922&sxsrf=AJOqlzUY2gj3jERTgXxJ-GLj7Xq71s_NeA:1676931957072&q=Procyanidin+A2&spell=1&sa=X&ved=2ahUKEwiGlfSskqX9AhU3FTQIHTByBUUQkeECKAB6BAgHEAE) | 285.44 | 285.44,289.27,449.00 | 4.89 ± 0.79 |

**Table S2**. MS parameters are used for the identification of metabolites derived from cranberry polyphenols present in serum and urine samples.

| **Compound** | **Molecular formula** | **[M-H]^-^** | **MS/MS fragments** | **Predicted MS/MS Spectrum*** |
| --- | --- | --- | --- | --- |
| ***Benzoic acid derivatives*** |  |  |  |  |
| Benzoic acid | C7H6O2 | 121 | 77.05, 92.04 | 121.02, 77.00, 103.01, 92.99 |
| 3-hydroxybenzoic acid | C7H6O3 | 136.91 | 93.07, 65.04 | 93.03, 137.02, 111, 65.00 |
| 4-hydroxybenzoic acid | C7H6O3 | 137.36 | 93.05, 65.05 | 137.02, 119.01, 93.03, 65.00 |
| 2-3 Dihydroxybenzoic acid | C7H7NO3 | 153.94 | 110.04, 92.04, 82.04 | 110.02, 92.99, 82.03, 66.03 |
| 2-5-Dihydroxybenzoic acid | C7H6O4 | 152.9 | 109.04, 108, 81.05 | 153.01, 109.02, 107.01, 80.99 |
| 3-4-dyhydroxybenzoic acid | C7H6O4 | 152.9 | 109.04, 81.04, 91.00 | 108.99, 80.99, 91.01 |
| Vanillic acid | C8H8O4 | 167 | 152.05,107.97 | 167.03, 151, 123.04, 107.01 |
| ***Phenylacetic acid derivatives*** |  |  |  |  |
| Homovanillic acid | C9H10O4 | 181.1 | 137.07, 121.99 | 181.05, 163.03, 137.06, 121.02 |
| Homovanillic acid sulfate | C9H10O7S | 260.95 | 181.07, 137.05, 121.97 | 181.05, 137.06, 122.03 |
| 3,4 dihydroxyphenyl acetic acid | C8H8O4 | 167.04 | 123.07 | 167.03, 149.02, 123.04 |
| 3-hydroxyphenyl acetic acid | C8H8O3 | 151 | 107.07, 65.07 | 151.03, 107.04, 65.03 |
| 4-Hydroxyphenyl acetic acid | C8H8O3 | 151.05 | 79.05, 107.07 | 109.02, 107.01 |
| ***Hippuric acid derivatives*** |  |  |  |  |
| Hippuric acid | C9H9NO3 | 177.95 | 134.13, 77.05, 56.00 | 134.06, 77.03, 56.01 |
| a-hydroxyhippuric acid | C9H9NO4 | 195.1721 | 94.07, 151.05 | 150.05, 94.03 |
| 4-Hydroxyhippuric acid | C9H9NO4 | 195.1721 | 150.05, 93.07 | 148.03, 95.02, 93.03 |
| ***Sinapic acid*** |  |  |  |  |
| p-coumaric acid | C9H8O3 | 162.94 | 119.00, 91.07, 93.07 | 119.05, 92.99, 89.03 |
| m-Coumaric acid 2022 | C9H8O3 | 162.95 | 119.00, 91.05, 93.07 | 119.04, 93.03, 89.03 |
| O-coumaric acid 2022 | C9H8O3 | 162.93 | 119.00, 117.04, 93.04 | 119.01, 117.03, 93.03 |
| ***Flavonol derivatives*** |  |  |  |  |
| Quercetin-3-O-ß-D-glucuronide | C21H18O13 | 477 | 301.05, 151.04, 179.04 | 301.03, 151.00, 175.02 |
| ***Valerolactone derivatives*** |  |  |  |  |
| 5'-(3',4'-Dihydroxyphenyl)-gamma-valerolactone sulfate | C11H12O7S | 287 | 207.11, 163.11, 122.00 | 207.06, 163.07, 147.04 |
| ***Cinnamic acid derivatives*** |  |  |  |  |
| Caffeic acid | C9H8O4 | 178.99 | 135.07, 107.07, 89.07 | 135.04, 107.04, 91.05 |
| Caffeic acid 3-O-b-D-glucuronide | C15H16O10 | 355.19 | 178.98, 135.13, 113.06 | 355.06, 179.03, 135.04, 131.035 |
| Caffeic Acid 4-O-b-D-glucuronide | C15H16O10 | 355.13 | 179.05, 135.05, 175.04 | 311.07, 293.06, 177.05, 175.02, 135.03 |
| Dihydro caffeic acid 3-O-sulfate | C9H6O7S | 261.06 | 181.13, 137.05, 109.07 | 179.03, 135.04, 96.96 |
| Ferulic acid | C10H10O4 | 193 | 134.04, 178.04, 149.13 | 193.10, 178.4, 149.3, 117.10 |
| Ferulic acid 4-O-B-D-Glucoronide | C16H18O10 | 369.05 | 193.04, 112.97, 175.04 | 193.05, 175.02, 103.04 |
| Dihydro ferulic acid 4-O-sulfate | C10H10O7S | 275 | 195.04, 136.04, 59.04 | 273.00, 193.05, 175.04, 96.96 |
| Dihydro ferulic acid 4-O-b-D-glucuronide | C16H20O10 | 371.08 | 195.14, 174.97, 112.97 | 371.09, 195.06, 175.02, 117.01 |
| Isoferulic acid 3-O-sulfate | C10H10O7S | 273.03 | 193.05, 134.07, 178.04 | 196.99, 179.03, 149.06 |
| Isoferulic acid 3-O-b-D-glucuronide | C16H18O10 | 369.14 | 193.05, 113.11, 178.10 | 193.03, 177.01, 149.06, 103.03 |
| Dihydro isoferulic acid 3-O-sulfate | C10H10O7S | 275 | 195.07, 136.05, 135.07 | 275.0, 195.06, 135.04, 121.02 |
| Dihydro isoferulic acid 3-O-ß-D-glucuronide | C16H18O10 | 371.13 | 195.07, 112.97, 175.04 | 195.06, 179.05, 125.05 |
| ***Catechol derivatives*** |  |  |  |  |
| Pyrochatechol-O-sulfate | C6H6O5S | 188.86 | 109.04, 79.99, 91.04 | 109.02, 79.01, 91.01 |
| ***Propionic acid derivatives*** |  |  |  |  |
| 3-(3-hydroxyphenyl) propionic acid | C9H10O3 | 164.96 | 121.00, 119.04, 106.07 | 121.06, 119.01, 105.03 |
| 3-(4-hydroxyphenyl) propionic acid | C9H10O3 | 164.92 | 121.04, 119.04 | 121.06, 119.05, 105.03 |

Table S3. Pharmacokinetic parameters of serum polyphenol metabolites following cranberry or placebo juice consumption

| **Metabolites** | **Session** | **AUC (nM*h)** | **C_max_ (nM)** | **T_max_ (h)** | **n** |
| --- | --- | --- | --- | --- | --- |
|  |  | **Placebo group** | | |  |
| Catechol-O-sulfate | Week 0 | 543801.20 ± 157739.29* | 97434.91 ± 78691.93 | 2.89 ± 3.89 | 9 |
|  | Week 6 | 425829.10 ± 125646.05 | 128610.95 ± 129869.99 | 2.00 ± 3.46 | 9 |
| 3-(3-hydroxyphenyl) propionic acid | Week 0 | 24352.41 ± 3371.34 | 3484.25 ± 502.72 | 1.78 ± .85 | 9 |
|  | Week 6 | 19284.99 ± 4122.61 | 3074.89 ± 690.84 | 2.22 ± 1.13 | 9 |
| 4-hydroxybenzoic acid | Week 0 | 4759.86 ± 2430.27 | 1673.65 ± 1013.53 | 3.20 ± 1.96 | 5 |
|  | Week 6 | 2509.65 ± 1330.96 | 1195.31 ± 662.82 | 0.67 ± .42 | 6 |
| 3-hydroxybenzoic acid | Week 0 | 1077.06 ± 415.07 | 397.77 ± 181.19 | 1.33 ± 1.33 | 6 |
|  | Week 6 | 607.03 ± 356.37 | 231.83 ± 103.77 | 0.29 ± .29 | 7 |
| 4-Hydroxyhippuric acid | Week 0 | 390.50 ± 296.93 | 135.95 ± 83.84 | 0.91 ± .73 | 11 |
|  | Week 6 | 59.24 ± 41.34 | 35.18 ± 25.25 | 0.57 ± .57 | 14 |
| **Metabolites** | **Session** | **Cranberry group** | | | **n** |
| Catechol-O-sulfate | Week 0 | 644329.43 ± 90216.66 | 148373.89 ± 114405.33 | 6.16 ± 3.05 | 25 |
|  | Week 6 | 667761.17 ± 151171.76 | 132743.37 ± 182900.75 | 3.48 ± 3.67 | 25 |
| 3-(3-hydroxyphenyl) propionic acid | Week 0 | 8255.50 ± 1915.58 | 1761.93 ± 359.27 | 4.67 ± 1.02 | 12 |
|  | Week 6 | 5873.46 ± 1701.77 | 1670.75 ± 461.42 | 2.67 ± .89 | 15 |
| 4-hydroxybenzoic acid | Week 0 | 12088.81 ± 9827.11 | 3109.88 ± 2348.03 | 2.72 ± .69 | 25 |
|  | Week 6 | 3745.53 ± 1678.13 | 1460.71 ± 704.32 | 2.17 ± .58 | 24 |
| 3-hydroxybenzoic acid | Week 0 | 6748.74 ± 5578.41 | 1735.47 ± 1329.16 | 2.35 ± .66 | 23 |
|  | Week 6 | 1828.74 ± 842.16 | 702.92 ± 350.64 | 2.25 ± .64 | 24 |
| 4-Hydroxyhippuric acid | Week 0 | 649.71 ± 376.88 | 152.47 ± 87.79 | 0.35 ± .16 | 23 |
|  | Week 6 | 1110.92 ± 614.00 | 305.11 ± 154.91 | 0.36 ± .17 | 22 |

Table S4. Urinary metabolite concentrations (nM) across post-consumption time windows (10–48h) at week 0 and week 6 of placebo juice intervention

| **Metabolites** | **Placebo group** | | | | |
| --- | --- | --- | --- | --- | --- |
| **Week 0** | **Baseline** | **10-18h** | **20-28h** | **30-38h** | **40-48h** |
| ***Hippuric acid derivatives*** |  |  |  |  |  |
| 4-Hydroxyhippuric acid* | 420.6 (254.8 - 586.4) | 179.7 (120.9 - 238.5) | 146.0 (94.1 - 197.8) | 215.9 (127.9 - 304.0) | 198.2 (116.9 - 279.6) |
| ***Cinnamic acid derivatives*** |  |  |  |  |  |
| Caffeic Acid 4-O-β-D-glucuronide | 5232.1 (-558.8 - 11023.1) | 1294.5 (422.4 - 2166.7) | 624.0 (102.0 - 1146.0) | 734.9 (193.9 - 1275.8) | 944.5 (89.9 - 1799.0) |
| Ferulic acid 4-O-sulfate | 767.4 (380.2 - 1154.7) | 412.6 (239.6 - 585.5) | 233.1 (138.3 - 327.9) | 599.5 (232.2 - 966.7) | 477.9 (54.8 - 900.9) |
| Dihydro ferulic acid 4-O-β-D-glucuronide | 585.6 (423.3 - 747.9) | 494.9 (336.4 - 653.4) | 474.7 (341.3 - 608.0) | 506.9 (352.9 - 660.9) | 471.8 (339.2 - 604.4) |
| Isoferulic acid 3-O-β-D-glucuronide | 9595.6 (5907.0 - 13284.1) | 4431.6 (2473.0 - 6390.3) | 4295.1 (2521.3 - 6069.0) | 5802.0 (1221.1 - 10382.9) | 4807.0 (2109.5 - 7504.5) |
| ***Catechol derivatives*** |  |  |  |  |  |
| Catechol-O-sulfate | 1284.6 (782.7 - 1786.5) | 366.5 (210.2 - 522.8) | 243.5 (164.2 - 322.9) | 556.6 (99.1 - 1014.2) | 395.6 (240.9 - 550.3) |
| **Week 6** | **Baseline** | **10-18h** | **20-28h** | **30-38h** | **40-48h** |
| ***Hippuric acid derivatives*** |  |  |  |  |  |
| 4-Hydroxyhippuric acid* | 290.5 (144.7 - 436.2) | 209.2 (137.8 - 280.5) | 257.7 (128.5 - 386.9) | 248.1 (101.2 - 395.1) | 212.0 (134.5 - 289.6) |
| ***Cinnamic acid derivatives*** |  |  |  |  |  |
| Caffeic Acid 4-O-b-D-glucuronide | 2198.8 (348.0 - 4049.5) | 603.8 (185.5 - 1022.1) | 685.1 (216.0 - 1154.2) | 875.1 (-244.5 - 1994.7) | 907.9 (-199.9 - 2015.7) |
| Ferulic acid 4-O-sulfate | 744.2 (373.5 - 1114.8) | 747.0 (343.9 - 1150.1) | 420.2 (259.2 - 581.2) | 816.3 (398.6 - 1234.1) | 465.5 (112.7 - 818.4) |
| Dihydro ferulic acid 4-O-b-D-glucuronide | 504.7 (341.1 - 668.4) | 494.5 (316.0 - 673.0) | 544.7 (393.3 - 696.0) | 499.8 (331.4 - 668.3) | 512.0 (358.0 - 665.9) |
| Isoferulic acid 3-O-b-D-glucuronide | 6387.0 (3349.2 - 9424.8) | 5683.1 (2736.9 - 8629.2) | 5692.6 (2959.7 - 8425.6) | 4145.1 (2568.8 - 5721.5) | 3598.4 (2335.2 - 4861.6) |
| ***Catechol derivatives*** |  |  |  |  |  |
| Catechol-O-sulfate | 1030.8 (520.1 - 1541.5) | 474.3 (230.2 - 718.4) | 536.6 (276.4 - 796.7) | 480.0 (265.9 - 694.1) | 505.8 (260.1 - 751.5) |

^β^ Values are mean (95% Confidence interval). ***P-value* < 0.05** was considered statistically significant. *Differences in metabolite concentration within cranberry and placebo treatment were detected using the Wilcoxon matched-pairs signed rank test. ^α^ 5-(3',4'-DP)-γ-v-4'-O-sulfate means 5-(3′,4′-dihydroxyphenyl)-γ-valerolactone-4'-O-sulfate *Values are presented as mean ± standard error.

Table S5. Urinary metabolite concentrations (nM) across post-consumption time windows (10–48h) at week 0 and week 6 of cranberry juice intervention

| **Metabolites** | **Cranberry group** | | | | |
| --- | --- | --- | --- | --- | --- |
| **Week 0** | **Baseline** | **10-18h** | **20-28h** | **30-38h** | **40-48h** |
| ***Hippuric acid derivatives*** |  |  |  |  |  |
| 4-Hydroxyhippuric acid* | 307.4 (172.7 - 442.2)^β^ | 281.5 (197.1 - 365.9) | 263.5 (197.3 - 329.6) | 337.9 (249.2 - 426.6) | 323.5 (229.4 - 417.7) |
| ***Cinnamic acid derivatives*** |  |  |  |  |  |
| Caffeic Acid 4-O-β-D-glucuronide | 1380.8 (754.1 - 2007.4) | 805.9 (204.2 - 1407.6) | 666.4 (261.0 - 1071.9) | 865.5 (180.2 - 1550.7) | 887.7 (270.8 - 1504.6) |
| Ferulic acid 4-O-sulfate | 698.3 (409.8 - 986.8) | 517.3 (347.5 - 687.1) | 329.6 (230.4 - 428.7) | 998.3 (371.4 - 1625.2) | 745.7 (110.6 - 1380.8) |
| Dihydro ferulic acid 4-O-β-D-glucuronide | 615.7 (440.4 - 790.9) | 501.8 (358.8 - 644.7) | 586.0 (469.1 - 703.0) | 591.9 (459.5 - 724.3) | 652.8 (510.1 - 795.5) |
| Isoferulic acid 3-O-β-D-glucuronide | 8045.7 (5469.4 - 10622.0) | 4049.9 (2902.5 - 5197.3) | 5207.7 (3155.9 - 7259.4) | 6454.1 (4523.3 - 8385.0) | 7080.0 (4511.6 - 9648.4) |
| ***Valerolactone derivatives*** |  |  |  |  |  |
| 5-(3',4'-DP)-γ-v-4'-O-sulfate^α^ | 884.5 (320.2 - 1448.7) | 136.2 (71.6 - 200.9) | 332.1 (129.4 - 534.9) | 180.4 (57.6 - 303.2) | 249.4 (150.6 - 348.3) |
| ***Catechol derivatives*** |  |  |  |  |  |
| Catechol-O-sulfate | 1025.5 (575.2 - 1475.8) | 1949.1 (976.9 - 2921.4) | 3166.8 (2117.2 - 4216.5) | 2270.3 (1381.0 - 3159.7) | 4306.1 (1278.4 - 7333.8) |
| **Week 6** | **Baseline** | **10-18h** | **20-28h** | **30-38h** | **40-48h** |
| ***Hippuric acid derivatives*** |  |  |  |  |  |
| 4-Hydroxyhippuric acid | 520.8 (375.2 - 666.3) | 482.5 (324.1 - 640.9) | 427.2 (288.6 - 565.9) | 402.6 (215.0 - 590.2) | 329.3 (210.2 - 448.4) |
| ***Cinnamic acid derivatives*** |  |  |  |  |  |
| Caffeic Acid 4-O-β-D-glucuronide | 1906.8 (660.8 - 3152.9) | 684.2 (203.3 - 1165.2) | 997.3 (151.9 - 1842.8) | 861.7 (166.8 - 1556.6) | 657.2 (68.5 - 1245.9) |
| Ferulic acid 4-O-sulfate | 1159.9 (579.7 - 1740.0) | 1052.3 (525.6 - 1579.0) | 819.7 (468.7 - 1170.8) | 842.8 (508.4 - 1177.3) | 629.6 (339.2 - 919.9) |
| Dihydro ferulic acid 4-O-β-D-glucuronide | 733.0 (558.4 - 907.6) | 602.7 (440.4 - 765.0) | 637.2 (491.7 - 782.8) | 659.4 (438.0 - 880.8) | 557.1 (444.1 - 670.1) |
| Isoferulic acid 3-O-β-D-glucuronide | 9148.7 (6852.6 - 11444.9) | 7968.4 (4584.8 - 11352.0)* | 7343.0 (5441.2 - 9244.9)* | 7243.4 (3467.1 - 11019.7) | 5897.1 (3908.6 - 7885.6) |
| ***Valerolactone derivatives*** |  |  |  |  |  |
| 5-(3',4'-DP)-γ-v-4'-O-sulfate | 987.1 (381.3 - 1592.8) | 265.3 (120.9 - 409.6) | 424.4 (228.3 - 620.5) | 332.3 (148.9 - 515.7) | 436.0 (129.4 - 742.5) |
| ***Catechol derivatives*** |  |  |  |  |  |
| Catechol-O-sulfate | 4606.0 (2956.7 - 6255.3)* | 3423.1 (2033.7 - 4812.4) | 4155.1 (3001.2 - 5309.0) | 3657.7 (2011.0 - 5304.5) | 3586.3 (2194.4 - 4978.2) |

^β^ Values are mean (95% Confidence interval). ***P-value* < 0.05** was considered statistically significant. *Differences in metabolite concentration within cranberry and placebo treatment were detected using the Wilcoxon matched-pairs signed rank test. ^α^ 5-(3',4'-DP)-γ-v-4'-O-sulfate means 5-(3′,4′-dihydroxyphenyl)-γ-valerolactone-4'-O-sulfate *Values are presented as mean ± standard error.

**Table S6.** ANOSIM and PERMANOVA statistics for gut microbiota beta diversity across Bray-Curtis, Weighted, and Unweighted UniFrac metrics

|  | **Test statistics** | **Bray-Curtis** | **Weighted UniFrac** | **Unweighted UniFrac** |
| --- | --- | --- | --- | --- |
| ANOSIM | R value | 0.048 | 0.073 | 0.036 |
|  | P value | 0.014 | 0.004 | 0.041 |
| PERMANOVA | P value | 0.296 | 0.026 | 0.051 |
